# Supplementary material for: Urolithin A Protects Porcine Oocytes from Artificially Induced Oxidative Stress Damage to Enhance Oocyte Maturation and Subsequent Embryo Development
Source: Int J Mol Sci. 2025 Mar 26;26(7):3037. doi: 10.3390/ijms26073037 (PMC11989139; doi:10.3390/ijms26073037)
Supplement: Supplementary file 1 [file ijms-26-03037-s001.zip › ijms-3495653-supplementary.pdf]

Urolithin A Protects Porcine Oocytes from Artificially Induced Oxidative Stress Damage to Enhances Oocyte Maturation and Subsequent Embryo Development

Wen Shi [1, †], Chaobin Qin[1, †], Yanyan Yang[1], Xiaofen Yang[1], Yizhen Fang[1], Bing Zhang[1], Dong Wang[1] ， Wanyou Feng[2, *] and Deshun Shi[1, *]

Table S1 Effects of Different dose of H_2O_2 Addition on Porcine Oocytes IVM

Concentration of H_2O_2 (μM)	No. of oocytes examined	% of Polar Body Extrusion(n)
Control	201	83.68±4.12% (168)^a
50	211	78.25±1.88% (165)^a
100	224	76.63±1.99% (173)^a
200	225	53.88±2.19 (121)^b
400	213	33.19±2.89% (71)^c

Data are the mean ± SEM. Values with different superscript letters within a column indicate significant differences ($P < 0.05$).

Table S2 Effects of Different dose of H_2O_2 Addition on Porcine Parthenogenetic Embryos Developmental Competence

Concentration of H_2O_2 (μM)	No. of embryos examined	% of cleavage (n)	% of blastocysts (n)
Control	238	87.96±4.30% (209)^a	34.83±1.30% (83)^a
50	245	87.57±2.35% (214)^a	31.31±0.80% (77)^{ab}
100	241	81.09±2.11% (195)^a	28.50±1.12% (69)^b
200	242	68.12±1.92% (165)^b	16.63±1.40% (40)^c
400	253	14.58±1.82% (37)^c	3.45±1.99% (8)^d

Data are the mean ± SEM. Values with different superscript letters within a column indicate significant differences ($P < 0.05$).

Table S3 Effects of Different dose of UA Supplement on H_2O_2-treated Porcine Oocytes IVM

**Concentration of H_2O_2 (μM)**	**No. of oocytes examined**	**% of Polar Body Extrusion(n)**
Control	246	85.99±2.13% (213)^{a}
0	274	51.67±3.33% (147)^{b}
5	179	68.15±2.94% (122)^{c}
10	173	76.15±1.26% (132)^{ac}
15	184	80.94±1.15% (149)^{a}
30	281	84.85±1.17% (239)^{a}
60	243	64.50±3.03% (156)^{c}
120	194	56.06±2.10% (108)^{b}

Data are the mean ± SEM. Values with different superscript letters within a column indicate significant differences ($P < 0.05$).

Table S4 Effects of Different dose of UA Supplement on H_2O_2-treated Porcine Parthenogenetic Embryos Developmental Competence

**Concentration of H_2O_2 (μM)**	**No. of embryos examined**	**% of cleavage (n)**	**% of blastocysts (n)**
Control	246	86.73±2.36% (214)^{a}	36.99±1.66% (92)^{a}
0	274	56.80±1.04% (156)^{b}	8.45±1.73% (26)^{b}
5	179	70.38±0.49% (126)^{c}	10.07±0.70% (18)^{b}
10	173	74.07±1.20% (128)^{cd}	12.64±1.19% (22)^{b}
15	184	78.74±0.97% (145)^{ac}	21.73±0.51% (40) ^{c}
30	227	81.92±1.40% (185)^{ad}	28.24±0.65% (64)^{d}
60	243	70.01±2.61% (171)^{c}	17.86±0.84% (43)^{c}
120	163	49.02±3.70% (80)^{b}	13.00±0.59% (21)^{b}

Data are the mean ± SEM. Values with different superscript letters within a column indicate significant differences ($P < 0.05$).

Table S5 Primer sequences used for qRT-PCR

Gene	Primer Sequences (5′ to 3′)	GenBank Accession No.
β-ACTIN	F: GATGACGATATTGCTGCGCT<br>R: TTCTCCATGTCGTCCCAGTT	XM 021086047.1
SIRT1	F: ATTCCAAGTTCCATACCCCAT<br>R: AGCTTGGCATATTCACCTCC	NM 001145750.2
PGC-1α	F: TGTGCAACCAGGACTCTGTA<br>R: GTCACTGCACCACTTGAGTC	NM 213963.2
SOD1	F: AGGATCAAGAGAGGCACGTTG<br>R: ACCTCTGCCCAAGTCATCTG	NM 001190422.1
CAT	F: GCTGAGTCCGAAGTCGTCTA<br>R: GTCAGGATATCAGGTTTCTGCG	NM 214301.2
GPX4	F: GGGCATGCTGGGAAATGCTATC<br>R: GTTGAGCTAGAGGTAGCACGG	NM 214407.1
AMPKα1	F：CGACAGCCGAGAAGCAGAAAC<br>R：TTGCCAACCTTCACTTTGCC	NM_001167633.1
Beclin	F: CTGCCGTTGTACTGTTCTGG<br>R: TGTCTCGCCTTTCTCAACCT	NM_001044530.1
LC3B	F: AACGAAATTCCTGGTGCCTG<br>R: TGGACACACTCACCATGCTA	NM_001190290.1
P62	F: ATAAGATCGCCCTGGAGTCG<br>R: TGGAGTTCACCTGTAGACGG	XM_005654936.2
Nanog	F: TCCACAAGCCCCAGAGTAAA<br>R: GCCTCTGAAATCTGTCGTTG	NM_001129971.2
Sox2	F: AAGAACAGCCCAGACCGAGT<br>R: CCGTCTCCGACAAAAGTTTCCAC	NM_001123197.1
Bax	F：GAGCAGATCATGAAGACAGGGG<br>R：AATGCGCTTGAGACACTCGC	XM_003127290.5
Bcl2	F：TTCTTTGAGTTCGGTGGGG<br>R：CCAGGAGAAATCAAATAGAGGC	XM_021099593.1

Table S6 Effects of UA Supplement on H_2O_2-treated Porcine In Vitro Fertilization Embryos Developmental Competence

Group	No. of embryos examined	% of cleavage (n)	% of blastocysts (n)
Control	305	67.27±6.14% (206)[a]	8.33±0.21% (21)[a]
H_2O_2	288	39.07±1.588% (112)[b]	2.90±1.01% (3)[b]
H_2O_2+UA	309	52.17±2.04% (159)[b]	3.93±0.57% (6)[b]

Data are the mean ± SEM. Values with different superscript letters within a column indicate significant differences ($P < 0.05$).

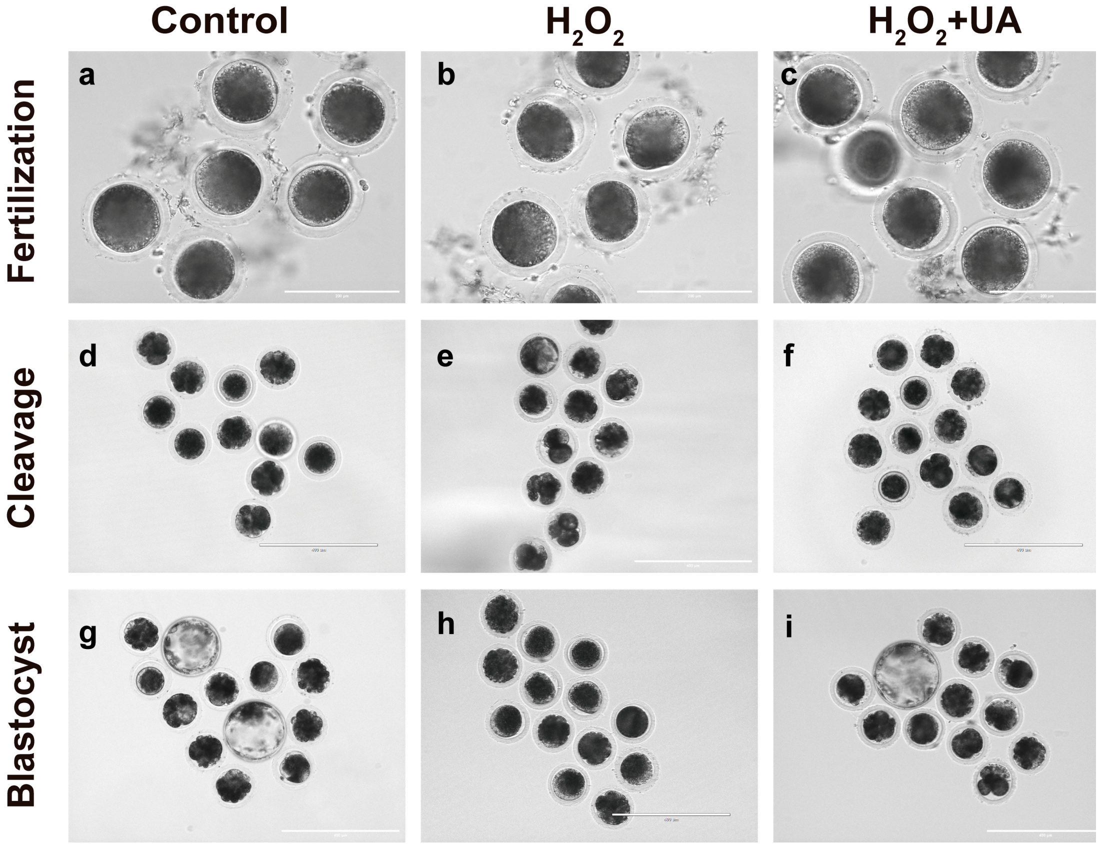

Figure S1 Representative images of UA Supplement on H_2O_2-treated Porcine In Vitro Fertilization. (a-c) Representative images of fertilization (Scale bar, 200 μm), (d-f) representative images of cleavage (Scale bar, 400 μm), (g-i) representative images of blastocysts (Scale bar, 400 μm).

Table S7 Effects of Different dose of UA Supplement on Porcine Conventional Oocytes IVM and Parthenogenetic Embryos Developmental Competence.

Concentration of UA (μM)	No. of embryos examined	% of Polar Body Extrusion(n)	% of cleavage (n)	% of blastocysts (n)
Control	178	81.29±1.05% (145)[abc]	82.69±1.04% (147)[a]	31.01±0.85% (55)[a]
5	179	81.71±0.82% (146)[abc]	80.89±0.67% (145)[a]	29.17±0.94% (52)[a]

10	152	80.77±2.51% (122)^{abc}	79.63±1.59% (121)^{a}	32.54±1.69% (49)^{ab}
20	150	84.97±3.37% (126)^{bc}	82.07±2.46% (122)^{a}	36.23±1.81% (54)^{b}
40	168	86.28±3.42% (144)^{bc}	86.07±2.16% (144)^{ac}	35.04±2.16% (58)^{ab}
80	178	74.37±1.53% (132)^{a}	75.59±1.90% (135)^{b}	29.84±1.41% (53)^{a}
160	153	77.23±1.28% (118)^{abc}	75.85±0.50% (116)^{a}	27.51±1.28% (42)^{a}

Data are the mean ± SEM. Values with different superscript letters within a column indicate significant differences ($P < 0.05$).
